# Epidemiological study of child and adolescent psychiatric disorders in Lithuania

**DOI:** 10.1186/s12889-018-5436-3

**Published:** 2018-04-24

**Authors:** Sigita Lesinskiene, Sigita Girdzijauskiene, Grazina Gintiliene, Dovile Butkiene, Dainius Puras, Robert Goodman, Einar Heiervang

**Affiliations:** 1 Faculty of Medicine, Institute of Clinical Medicine, Clinic of Psychiatry, Vilnius University, Vilnius, Lithuania; 2 Faculty of Philosophy, Institute of Psychology, Vilnius University, Vilnius, Lithuania; 3 King’s college London Institute of Psychiatry, Psychology and Neuroscience, London, United Kingdom; 4 Institute of Clinical Medicine, University of Oslo, Oslo, Norway

**Keywords:** Epidemiology, Child and adolescent psychiatric disorders, Prevalence, Risk factors

## Abstract

**Background:**

From the public health perspective, epidemiological data of child mental health and psychosocial correlates were necessary and very lacking in Lithuanian society that has been undergoing rapid socio-economic change since the past decades. Together with determining the prevalence rates of disorders and assessing the needs for the services, this study has also shifted attention from the highly selective samples of children attending children and adolescent mental health services towards less severe cases of psychopathology as well as different attitudes of parents and teachers. The aim of the first epidemiological study in Lithuania was to identify the prevalence of psychiatric disorders in the community sample of children.

**Methods:**

Child psychiatric disorders were investigated in a representative sample of 3309 children aged 7–16 years (1162 7–10-year-olds and 2147 11–16-year-olds), using a two-phase design with the Lithuanian version of the Strengths and Difficulties Questionnaire (SDQ) in the first screening phase, and the Development and Well-Being Assessment (DAWBA) in the second diagnostic phase.

**Results:**

The estimated point prevalence of ICD-10 psychiatric disorders was 13.1% for the total sample (14.0% for the child sample and 12.1% for adolescent sample). The most common groups of disorders were Conduct disorders 6.6% (7.1% for child sample and 6.0% for adolescent sample), Anxiety disorders 5.0% (5.9% for child sample and 6.0% for adolescent sample), with Hyperkinesis being less common 2.0% (2.7% for child sample and 1.2% for adolescent sample). Potential risk factors were related to individual characteristics of the child (gender, poor general health, and stressful life experiences), and the family (single parenthood, foster care, unfavourable family climate, disciplining difficulties, worries related to TV or computer use).

**Conclusions:**

The overall prevalence of youth psychiatric disorders was relatively high in this representative Lithuanian sample compared to Western European countries. The SDQ and DAWBA measures appear useful for the further research and clinical practice in this society.

## Background

This study represents the effort of an international collaboration in conducting the first epidemiological study of youth mental health in Lithuania. After Lithuania regained its independence in 1990, the transitional period in political, economic and social systems was characterized by growing concern about the mental health of children and adolescents, including high rates of suicide, deliberate self-harm, juvenile delinquency, and drug and alcohol abuse [1–4]. Despite its necessity in a society undergoing socio-economic change, epidemiological data on youth mental health and psychosocial correlates have been lacking.

Lithuania is an East European country with a total population of around 3 million, of whom approximately 560,300 are aged from 7 to17 years. Children and adolescent mental health problems often have serious long-term debilitating effects [5–7]. Early identification and treatment of these problems are in the best interest of children, adolescents, their families and society as a whole [8–11]. All three essential ways in which epidemiology can contribute to our understanding of children and adolescent mental health: community burden, measurement, and triage [12] were of utmost importance planning and conducting the survey.

In this study we present the first large-scale survey of child and adolescent psychiatric disorders in the Baltic countries and post-soviet Eastern European countries. For adequate planning of services, including evidence-based mental health prevention and intervention, a population-representative survey of children and adolescent estimating mental disorders was urgently needed. The aim of the this study was to estimate the prevalence of ICD-10 psychiatric disorders in the community sample of schoolchildren in Lithuania, addressing prevalence, comorbidity and associated risk factors.

## Methods

### Sample

According to the Law on Education Republic of Lithuania the primary education curriculum shall start in that calendar year when child turns 7 year. A child under 16 years of age cannot terminate studies in compulsory education programs and must study according to primary and basic education curricula. The target group included children and adolescents aged 7 to 16 years. A national representative sample was selected by stratified sampling of subjects from urban, town and rural schools (see Table 1). One hundred and seventy selected classes from 15 urban, 10 town and 22 rural schools throughout the country were included. Questionnaires about 3309 children (1162 7–10-year-olds and 2147 11–16-year-olds) were obtained.

**Table 1:** Demographic characteristics of Lithuanian epidemiological study sample (*n* = 3309)

	7–10 year		11–16 year		Total	
	N	%	N	%	N	%
Gender						
Male	597	51.4	1102	51.3	1699	51.3
Female	565	48.6	1045	48.7	1610	48.7
Area of residence						
Urban	445	38.3	899	41.9	1344	40.6
Town	373	32.1	600	27.9	973	29.4
Rural area	344	29.6	648	30.2	992	30.0

### Instruments and procedure

The study conducted during the years 2004–2007 consisted of a screening questionnaire phase and a diagnostic interview phase.

#### The screening phase

The screening measure was the Lithuanian version of the Strengths and Difficulties Questionnaire (SDQ) with Impact supplement [13, 14] (www.sdq.info). The SDQ asks about 25 attributes, some positive and some negative, identical for parent and teacher versions. The items are divided into five scales of five items each, generating scores for Emotional symptoms, Conduct problems, Hyperactivity, Peer problems, and Prosocial behaviour. The Impact supplement assesses distress to the child, interference with everyday life, and burden for others [14]. The SDQ has shown acceptable reliability and validity, performing at least as well as lengthier and longer-established alternatives [15]. An important initial part of the current study was translation and establishment of norms for the SDQ in Lithuania. All three SDQ versions (parent, teacher and self-report) were translated into Lithuanian, followed by psychometric analyses. Results for internal consistency, inter- and intra-scale correlations, exploratory and confirmatory factor analyses, comparison with clinical groups, and inter-rater correlations, indicated adequate psychometric properties [16].

Questionnaires were completed by 3284 (99.2%) teachers and 3052 (92.2%) parents. Responses from teachers only were obtained for 47 (2.2%) children and adolescents. Out of 2147 adolescents aged 11–16 years, 1948 (90.7%) completed the self-report version of the SDQ. Data about 1858 (86.5%) adolescents were obtained from all three types of informants: parents, teachers and adolescents. Out of 1162 children aged 7–10 years, there were 1144 (98.5%) sets of data both from parents and teachers.

A computerized algorithm for the prediction of psychiatric disorders from multi-informant SDQ data was used [17]. The predictive algorithm generates “unlikely”, “possible” or “probable” ratings for four broad categories of disorder, namely Conduct disorders, Emotional disorders, Hyperactivity disorders, and Any psychiatric disorder (http://www.sdqinfo.com/c4.html). Cut-off on SDQ scores used in a computerised algorithm were obtained from Lithuanian SDQ norms [16]. Predictions of Any disorder for the child sample, adolescent sample, and the total sample are shown in Table 2.

**Table 2:** Predictions of diagnosis according SDQ-algorithm

	7–10 year		11–16 year		Total	
	N	%	N	%	N	%
Any diagnosis						
Unlikely	773	66.5	1196	55.7	1969	59.5
Possible	259	22.3	666	31.0	925	28.0
Probable	130	11.2	285	13.3	415	12.5

**Fig. 1:**
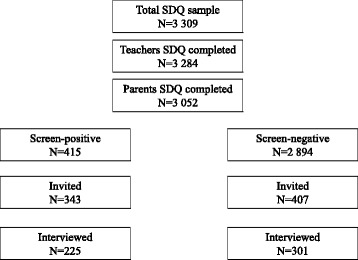
Flow chart of screening and diagnostic interview phases

In the current study, ‘Unlikely’ and ‘Possible’ were classified as screen-negative, while ‘Probable’ subjects were classified as screen-positive. Screen-positive subjects and a random 15% of screen-negative subjects were invited to take part in the second diagnostic interview phase (Fig.1).

#### The diagnostic interview phase

Teachers and parents of included subjects were interviewed with the DAWBA. The DAWBA is a package of questionnaires, interviews and rating techniques designed to generate ICD-10 and DSM-IV psychiatric diagnoses for 5–16-year-olds (www.dawba.com). In this study we used regular operationalised diagnoses that were included in DAWBA by author prof. R. Goodman [18]. During the time of conducting the survey, the author of DAWBA prof. R. Goodman has elaborated and added to DAWBA a new section for the Depression.

The DAWBA combines the features of structured and semi-structured interviews, with open-ended questions added if symptoms in an area are indicated in the structured part. As for the SDQ, the DAWBA was translated into Lithuanian and independently back-translated to check its fidelity, with approval of the final version by the developer of the DAWBA, R. Goodman. A computerized scoring program for the DAWBA integrates data from all informants (parents, teacher and youth). Answers to open-ended questions were translated into English before diagnostic rating was performed by R. Goodman and E. Heiervang, both experienced raters with demonstrated high inter-rater agreement.

A Family Background Questionnaire for parents was developed by the authors. It consisted of 19 questions about family structure, family relations, child mental and somatic health, school environment, out-of-school activities, TV and computer time. A Supplementary Questionnaire for teenagers, also developed by the authors, had 28 questions about leisure time activities, school grades, daily routines, duties at home, friends, pets, sense of happiness, and hopes for the future.

For the child sample, 116 screen-positives and 137 screen-negatives were invited. There were 35 (13.8%) non-respondents, resulting in a final child sample of 96 screen-positives and 122 screen-negatives.

For the adolescent sample, 227 screen-positives and 270 screen-negatives were invited. However, there were 189 (38%) non-respondents, resulting in a final adolescent sample of 129 screen-positives and 179 screen-negatives. Characteristics of the child and adolescent samples are presented in Table 3.

**Table 3:** Demographic characteristics of DAWBA interview phase samples (*n* = 526)

	Child sample (7–10 years)		Adolescent sample (11–16 years)		Total	
	No	%	No	%	No	%
Gender						
Male	136	62.4	147	47.7	283	53.8
Female	82	37.6	161	52.3	243	46.2
Area of residence						
Urban	87	39.9	125	40.6	212	40.3
Town	65	29.8	77	25.0	142	27.0
Rural area	66	30.3	106	34.4	172	32.7

### Statistical analysis

To estimate weighted prevalence rates for disorders, probabilities were calculated. The weights reflect the number of individuals in the first phase that each record in the second phase represents. Weights were calculated both for the child sample (*n* = 1107) and adolescent sample (*n* = 2202). Logistic regression analyses were performed using each diagnosis (0 = no, 1 = yes) as the dependent variable, and risk factors as independent variables for the child and adolescent samples.

## Results

### Response bias

Response bias rates are presented in Table 4. Whereas 99.3% of the population was assessed by teacher SDQ, 93.6% of these children’s parents also completed questionnaires. Comparing the 2979 subjects with SDQ data from both teachers and parents with the 203 subjects with teacher data only, the latter group had significantly higher teacher-reported psychopathology (teacher SDQ total mean score 11.3 (SD 6.5) versus 8.9 (SD 6.6); *t* = 5, df = 3180, *p* < 0.001).

**Table 4:** Response bias rates of participants and nonparticipants of parent and teachers SDQ for child and adolescent sample screen-positive and screen-negative cases

	Screen-positive		Screen-negative	
	Child sample (7–10 years)			
	Participants (*n* = 85)	Nonparticipants (*n* = 19)	Participants (*n* = 114)	Nonparticipants (*n* = 13)
Parent SDQ total score mean (SD)	19.1 (5.3)*	16.2 (5.4)	11.2 (6.3)	11.4 (4.9)
Teacher SDQ total score mean (SD)	18.1 (8.0)	19.9 (5.9)	8.1 (6.6)	7.6 (5.8)
	Adolescent sample (11–16 years)			
	Participants (*n* = 125)	Nonparticipants (*n* = 97)	Participants (*n* = 165)	Nonparticipants (*n* = 85)
Parent SDQ total score mean (SD)	17.5 (6.4)	17.8 (6.2)	10.1 (5.1)	10.8 (5.5)
Teacher SDQ total score mean (SD)	15.4 (7.6)	15.4 (7.6)	7.7 (5.5)*	9.2 (6.0)

*p < .05

Analysis of response bias revealed higher parent SDQ mean scores for screen-positive participants in child sample and higher teacher SDQ mean scores for screen-negative participants in adolescent sample.

From the invited sample of 116 screen-positives children, 96 (82.8%) participated in the DAWBA interview phase. From the invited sample of 137 screen-negatives children, 122 (89.1%) participated. From the invited sample of 227 screen-positives adolescents 129 participated in the DAWBA interview phase (47.8%). From the invited sample of 270 screen-negatives adolescents, 179 (66.3%) participated.

There were no significant differences between participants and nonparticipants regarding age or gender for screen-positives and screen-negatives.

### Prevalence rates

Weighted prevalence estimates for the main ICD-10 diagnostic groups in children and adolescents are shown in Table 5. The prevalence rate was 14.0% for any psychiatric diagnosis in the child sample and 12.1% in the adolescent sample. The most frequent groups of disorders were Conduct disorders (7.1% for child sample and 6.0% for adolescent sample), and Anxiety disorders (5.9% and 4.1% respectively). Among the less prevalent disorders were Hyperkinesis, Tic disorder and Autistic disorder (for both sample groups), and Depressive disorder (assessed in the adolescent sample only). No subjects were diagnosed with Panic disorder, Agoraphobia, Obsessive compulsive disorder or Post-traumatic stress disorder.

**Table 5:** Prevalence rates (95% confidence interval) for ICD-10 mental disorders

	Child sample (*n* = 1107)	Adolescent sample (*n* = 2202)
Any disorder	14.0 (12.1–16.2)	12.1 (10.8–13.6)
Conduct	7.1 (5.7–8.8)	6.0 (5.1–7.1)
Anxiety	5.9 (4.6–7.4)	4.1 (3.3–5.0)
Depression	Not assessed	2.4 (1.8–3.2)
Hyperkinesis	2.7 (1.9–3.8)	1.2 (0.9–1.8)
Tic disorder	1.0 (0.1–1.8)	0.7 (0.4–1.2)
Autistic disorder	0.9 (0.1–1.7)	0.1 (0.0–0.4)

### Comorbidity

14% of child sample had DAWBA diagnosis (see Table 5). 29% of them had one or more comorbid disorders; varying from 17% for Anxiety disorder to 38% for Conduct disorder, and 83% for Hyperkinesis. In the adolescent sample 12.1% have DAWBA diagnoses (see Table 5) and 23% of them had one or more comorbid disorders; varying from 23% for Emotional disorders (Depression or Anxiety), to 36% for Conduct disorders and 100% for Hyperkinesis.

### Risk factors

Correlates of disorders are presented in Table 6. Gender as risk factor played a different role in child and adolescent samples. Conduct and any ICD-10 disorder were significantly more common in boys compared to girls, while teenage girls outnumbered boys for Emotional disorders.

**Table 6:** Unadjusted OR of analyses of children and family correlates for the main ICD-10 categories

	Any ICD-10 disorder	Emotional disorder	Conduct disorder	Hyperkinesis
Child sample *n* = 218				
Girls	0.5* (0.2–0.9)	1.8 (0.8–4.2)	0.2* (0.1–0.5)	0.2 (0.1–1.1)
Poor general health	8.7*** (2.3–32.6)	2.6 (0.7–10.3)	4.5** (1.4–14.1)	7.8** (2.1–29.5)
Children with SEN	3.7** (1.6–8.9)	2.4 (0.8–7.2)	2.1 (0.8–5.4)	3.3 (1.0–11.4)
Excessive TV or computer use	2.0* (1.1–3.5)	1.3 (0.6–3.1)	2.1* (1.1–4.3)	9.5** (2.1–43.3)
Discipline problems	3,5*** (1.9–6.5)	1.1 (0.4–2.7)	4,5*** (2.2–9.3)	7.7*** (2.4–25.3)
Adolescent sample *n* = 308				
Female gender	1.0 (0.6–1.7)	3.3* (1.3–8.6)	0.4 (0.2–1.0)	0.2 (0.0–1.5)
Single parent or foster home	1.3* (1.0–1.8)	1.3 (0.9–1.8)	1.4 (1.0–1.9)	0.5 (0.1–2.7)
Good family climate	0.3*** (0.2–0.5)	0.4** (0.2–0.7)	0.3*** (0.1–0.5)	0.3* (0.1–0.9)
Children with SEN	3.4* (1.2–9.3)	1.5 (0.3–6.8)	2.7 (0.8–8.9)	3.5 (0.4–31.9)
Excessive TV or computer use	1.8* (1.0–3.3)	2.5* (1.1–5.6)	1.2 (0.6–2.5)	3.5 (0.6–19.1)
Discipline problems	8.6*** (4.5–16.5)	3.2** (1.4–7.3)	12.2***(5.4–27.7)	8.0* (1.4–44.7)
Likes school	0.3** (0.1–0.6)	0.3* (0.1–0.8)	0.2*** (0.1–0.5)	0.2* (0.0–0.8)
Learning problems	2.3*** (1.6–3.3)	2.4** (1.4–3.9)	2.0** (1.3–3.1)	2.8* (1.0–7.6)
Good grades in Lithuanian	0.7** (0.5–0.9)	0.7 (0.5–1.1)	0.6** (0.4–0.9)	0.5 (0.2–1.2)
Good grades in Mathematics	0.6*** (0.4–0.8)	0.8 (0.5–1.2)	0.5** (0.3–0.8)	0.2 (0.1–0.8)

*p < .05, ** *p* < .01, *** *p* < .001

In child sample poor general health, excessive TV or computer use and having discipline problems were significantly associated with Conduct disorder, Hyperkinesis and any ICD-10 disorder. Children with special educational needs (SEN) were more likely to have any ICD-10 disorder.

In adolescent sample single parent family or foster home, bad family climate, learning problems, having SEN, poor grades in school, discipline problems and excessive TV or computer use had significant higher rates of any ICD-10 diagnosis. Being the girl, having learning problems and dislike of the school, as well as poor family climate, excessive TV or computer use and discipline problems were significantly associated with emotional disorder.

## Discussion

This is the first study in the Baltic countries based on a large representative sample from the general school population determining the prevalence rates of mental disorders. The overall prevalence for ICD-10 psychiatric disorders in the combined population of 7–16-year-olds of 13.1% corresponds well with the pooled global prevalence of 13.4% (CI 95% 11.3–15.9) for children and adolescents presented in a recent meta-analysis of studies in children and adolescents [19]. We here also report some distinct differences between children and adolescents for the prevalence of disorders, with a higher overall prevalence in children aged 7–10 years compared to adolescents aged 11–16 years. This difference is mainly accounted for by higher prevalence of Hyperkinesis and Conduct disorders in the younger age group.

Many studies from different countries and parts of the world have used SDQ and DAWBA, allowing for cross-cultural comparisons. The results from the present study can therefore be compared with the results from epidemiological studies that have been used similar measures of psychopathology. The 95% confidence interval for the prevalence of psychiatric disorders in Lithuania (12–16% for 7–10-year-olds and 11–14% for 11–16-year-olds) is close to what was reported from Yemen (12–20% for 7–10-year-olds) [8], Bangladesh (11–21% for 5–10-year-olds) [20], Russia (10–20% for 7–14-year-olds) [21], Omani (13.9% for 14–23-year-olds) [22], Israeli (11.7% for 14–17-year-olds) [23], and Australia (13.9% for 4–17-year-olds) [7]. However, lower prevalence has been reported from Britain (9–10% for 5–15-year-olds) [24] and Norway (6–9% for 8–10-year-olds) [25]. These comparisons suggest that Lithuanian rates of psychopathology are generally in line with what has been reported by studies which used the same measures in other low- or middle-income countries, but higher than what has been reported in high-income countries.

The estimated overall prevalence of 13.1% reported here also corresponds well with the rate of 12.5% for ‘probable diagnosis’ predicted from the multi-informant SDQ-algorithm. Although the SDQ as a screening instrument is much shorter than the DAWBA, it seems to provide a reliable and effective overall estimate for mental disorders in the population. This is supported by other studies, showing that predictions based on multi-informant SDQs may provide a cheap and quick way for estimating the prevalence rates of mental disorders in children and adolescents [26–28].

Furthermore, several family and environmental characteristics were examined to explore their association with the prevalence of mental disorders. In both groups disciplining difficulties reported by parents were prominent. The results of the study revealed some differences in potential risk factors between child and adolescent samples. In the child sample the association of poor general health with mental health disorders was one of the strongest and could be interpreted as potential risk factor. This is in line with other studies showing that poor mental health in childhood is strongly related to other health and development concerns [29–31]. In the adolescent sample we were able to evaluate more potential risk factors than in the child sample. As shown by others [6, 31, 32], a number of family factors were associated with adolescent mental health problems. In the present study this included single parenthood, unfavourable family climate and disciplining difficulties. Another important group of the potential risk factors in the adolescent sample was related with the school context. Our results confirm the evidence that youth with mental health problems perform less well in school and attain lower levels of education than other youth [6, 33, 34]. We also find learning difficulties and dislike of school to be associated with Conduct disorders, Emotional disorders and Hyperkinesis, while low achievement shows a strong association with Conduct disorders.

### Strengths and limitations

A great advantage of this study was the international cooperation and support of colleagues with a long-standing experience in epidemiological studies. This study is a population-representative survey of children and adolescent in Lithuania and is not reliant on clinical data which may underestimate the prevalence of mental disorders. Other important strength of the study is high response rates obtained from three sources of informants. Participation of teachers (99.2%) and parents (92.2%) was achieved as a result of active communication with schools and a well-organized information process that included well-designed information letters. By determining prevalence rates of multiple mental disorders, the study may contribute to a shift of attention from highly selective samples of children with severe disorders, towards the more prevalent mental health problems encountered in Lithuanian children and adolescents. The study also covered a wide age range from 7 to 16 years. Similarly to more recent psychiatric epidemiological studies [7, 22, 23, 28, 35, 36], the study included adolescents as an informant group. Data were collected with the use of Lithuanian versions of the highly validated measures of psychopathology; SDQ and DAWBA, and diagnostic rating by experienced experts.

The study has also some important limitations. First, dropouts from the DAWBA interview phase were not reached and surveyed. This could give an impression of somewhat incomplete data in the interpretation of risk factors presented in this study. Also, the data about family SES and educational level of parents who participated in the second phase of the survey were not obtained sufficiently. Presumably, parents with higher education were more willing to participate in this survey than those with lower education and SES. Second, the relatively low prevalence reported for Hyperkinesis, Autistic disorder and Depression. The adolescent sample was interviewed additionally with the newly developed Depression Section of DAWBA. Indeed, we suggest that a further dynamic initiative and undertaking could focus on the development of instruments for teenagers that would capture their peculiarities and complex comorbidities. Third, data on the risk factors were obtained by non-standardized questionnaires with a cross-sectional design; it was no possibility to separate causal risk factors from psychosocial consequences of the disorders.

### Implications

Our findings suggest that improvement of the general health, family conditions and school environment may be beneficial for CAMH. School age is a critical period with tremendous changes in the child’s environment, demands and rapid development of cognitive and social abilities. Early identification of difficulties gives the opportunity to offer relevant interventions, in order to assure successful adaptation to school [31].

The burden of mental illness has been shown to be substantial not only for the individual, but also for the family and the society [6, 9, 37]. As identified by the 2010 Global Burden of Disease Study, nationally representative studies into the prevalence of mental disorders in children and youth aged 0–24 years are scarce in most parts of the world [38]. Consequently, for many countries mental disorders will remain invisible or will be viewed as a low priority compared to other major global health agendas [39, 40].

Although most regions of Lithuania provide child and adolescent psychiatric outpatient services, a stronger focus on quality and effectiveness of services is needed in most CAMH organizations. Also, there is a need for services and specialized clinical programs adjusted to the child’s age and disorder. As for specialized treatment, the creation of services also targeting prevalent problems like anxiety and conduct disorders seems important in order to improve the mental health of the youth population.

Dissemination of the results of this study and description of the survey background and its process allow further promotion of CAMH and enhance the awareness of the need for adequate mental health services for children and adolescents. Regular mental health investigations and cross-cultural comparisons in this area of research should be further developed.

## Conclusions

SDQ and DAWBA appear to be sensitive and useful tools for further research and clinical practice; moreover, they allow for reliable cross-cultural comparisons. The overall prevalence of 13.1% of ICD-10 psychiatric disorders was in line with findings from other low and middle income countries, but higher than what has been reported from high income European countries. Knowing the prevalence of mental disorders among children and adolescents in Lithuania is important for the development of evidence-based CAMH services. Analysis of the risk factors reveals that issues concerning the general health and family and school environment are of high importance in the treatment of mental health disorders in children and adolescents.

## Abbreviations

CAMH: Child and Adolescent Mental Health
DAWBA: Development and Well-Being Assessment
SDQ: Strengths and Difficulties Questionnaire
